# Virtual Reality and Lower Limb Rehabilitation: Effects on Motor and Cognitive Outcome—A Crossover Pilot Study

**DOI:** 10.3390/jcm11092300

**Published:** 2022-04-20

**Authors:** Augusto Fusco, Silvia Giovannini, Letizia Castelli, Daniele Coraci, Dario Mattia Gatto, Giuseppe Reale, Roberta Pastorino, Luca Padua

**Affiliations:** 1 UOC Neuroriabilitazione ad Alta Intensità, Fondazione Policlinico Universitario A. Gemelli IRCCS, 00168 Rome, Italy; augusto.fusco@policlinicogemelli.it (A.F.); dario.gatto90@gmail.com (D.M.G.); giuseppe.reale@policlinicogemelli.it (G.R.); luca.padua@unicatt.it (L.P.); 2 Department of Geriatrics and Orthopaedics, Università Cattolica del Sacro Cuore, 00168 Rome, Italy; silvia.giovannini@unicatt.it; 3 UOS Riabilitazione Post-Acuzie, Fondazione Policlinico Universitario A. Gemelli IRCCS, 00168 Rome, Italy; 4 Department of Aging, Neurological, Orthopaedic and Head-Neck Sciences, Fondazione Policlinico Universitario A. Gemelli IRCCS, 00168 Rome, Italy; 5 Dipartimento di Neuroscienze, Università di Padova, 35128 Padova, Italy; danielecoraci@aol.com; 6 Department of Neurosciences, Università Cattolica del Sacro Cuore, 00168 Rome, Italy; 7 Department of Woman and Child Health and Public Health—Public Health Area, Fondazione Policlinico Universitario A. Gemelli IRCCS, 00168 Rome, Italy; roberta.pastorino@policlinicogemelli.it

**Keywords:** severe acquired brain injury, traumatic, virtual reality, cognitive function, rehabilitation, disability, lower limb, personalized medicine

## Abstract

The effectiveness of virtual reality (VR) in the motor and cognitive rehabilitation of patients with severe acquired brain injury (sABI) is unclear. This randomized, controlled, crossover, single-blinded, pilot study investigates the cognitive and motor effects of lower limb robotic therapy with and without VR visual feedback in a group of patients with ABI. A total of 23 patients with ABI were randomized into two groups: one group (VR-NVR) underwent a 2-week rehabilitation for the lower limbs training with a robotic device (Omego^®^) with VR feedback, followed by 2 weeks without VR; the other group (NVR-VR) performed the protocol in the opposite order. Patients were evaluated at baseline, after two and four weeks of treatment using the Level of Cognitive Functioning scale (LCF), Disability Rating Scale (DRS), and Motricity Index for Lower Limb (MI-LL) in the most affected limb. At the end of the intervention, both groups significantly improved in all the outcomes. A significant difference was found between VR treatment versus non-VR treatment for LCF (*p* = 0.024) and for DRS (*p* = 0.043) after the second week, while no significant differences were found in the group NVR-VR at T1. Our study indicates how the combination of robotic treatment with VR is effective in enhancing the recovery of cognitive function in patients with ABI, also improving disability and muscular function. Further, VR seems to enhance the early recovery process of motor and cognitive functions.

## 1. Introduction

Traumatic and non-traumatic severe acquired brain injury (sABI) are determined by damage to the brain of sudden or rapid onset, causing a temporary or permanent change in mental status as determined by the Glasgow Coma Scale [1]. It represents a substantial cause of morbidity and mortality in Western countries [2]. The clinical features of these patients are heterogeneous, ranging from mild symptoms to long-lasting cognitive and/or motor deficits [1,3]. Due to the uncertain functional and life prognosis of the patients, adequate rehabilitative treatments should be carried out early, from the first days of admission to the intensive care unit, to facilitate physical reconditioning and the neuroplasticity process of the central nervous system [4,5].

Rehabilitation of patients with sABI requires a combination of personalized interventions, including motor treatments, occupational therapy, speech therapy, neuropsychological and cognitive treatments [6]. Previous studies highlighted how rehabilitation must be guided by motor learning principles in order to remodel disrupted neural circuitries and allow voluntary motor activation restoration [7,8,9,10]. The treatments should encompass motor-cognitive skills in order to facilitate the recovery of global functioning in individuals with severe disability [11].

Many technological therapeutic approaches, such as robotic therapy, peripheral or brain electrical stimulation, and virtual reality (VR), have been proposed [12,13,14]. These devices increase the effectiveness of rehabilitation with restorative or adaptive purposes, both as add-on or stand-alone therapeutic interventions [15].

Treatments based on VR offer the opportunity to boost motor rehabilitation programs, facilitating the physiological activation of brain areas dedicated to motor learning. Enriched VR training can be used to provide a repetitive practice with multisensory stimuli (audio, visual, motor, proprioceptive), maximizing processes of neuroplasticity and thus motor learning and recovery [16]. The use of integrated rehabilitation therapy has proven to produce better results than a single-mode treatment, with a progressive and overall improvement in terms of sensorimotor performance [17]. The visual feedback is able to activate a network of cortical regions, including the superior and inferior parietal cortices, ventral premotor cortex, primary motor cortex, dorsal premotor cortex, inferior frontal gyrus and supplementary motor areas [18]. Continuous training with multisensory stimuli can produce long-term effects on brain excitability and neuronal plasticity [19], enhancing treatment efficacy [20]. This activation of the neural system is the basis for motor learning, including complex motor tasks such as locomotion [12,21].

VR-based technologies are demonstrating to be a valid tool to increase patient engagement across a wide number of diseases [22]. In patients with stroke and Parkinson’s disease, VR systems have shown promising results for gait and balance recovery [23,24]. An updated Cochrane review has also found a beneficial effect of VR in improving upper limb function and daily life activities when used in addition to usual care (increasing overall therapy time) in people with stroke, even if no significant evidence has been found on gait speed, balance, participation, or quality of life [25].

Despite these advantages, the use of VR technology remains limited in the rehabilitation of patients with sABI. In studies with a small number of enrolled patients with mild traumatic brain injury, its use was found to produce moderate improvement in terms of gait and balance [26,27]. Notwithstanding the huge number of individuals, patients with sABI enrolled in scientific protocols are still few. This could be due to the too severe conditions, limiting adherence to the rehabilitation program. The cognitive features of patients with sABI may influence the overall effectiveness of VR treatments, limiting its use in daily practice. Conversely, the possible limitations to the implementation of treatments with VR could be overcome in intensive hospital settings due to multidimensional therapeutic approaches. Therefore, it is necessary to provide more relevant data for using VR in the rehabilitation of patients with sABI.

The aim of this study was to investigate the cognitive and motor outcomes of lower limb robotic therapy with and without VR in a group of patients with severe acquired brain injury (traumatic and non-traumatic). We hypothesized that the visual feedback provided by VR, in addition to robotic therapy, could be more beneficial at improving patients’ cognitive and motor functions than conventional robotic therapy alone.

## 2. Materials and Methods

This is an interventional, randomized, controlled, crossover, single-blinded study.

Patients with severe acquired brain injury (sABI) that matched the following inclusion criteria were included: disease onset within the last six months, Trunk Control Test (TCT) value ≥ 36, and Level of Cognitive Functioning (LCF) ≥ 3. Exclusion criteria were the following: clinical instability, severe visual impairment (central or peripheral, acquired before or after the acute event), severe cognitive impairment or severe apraxia, and inability to understand and sign informed consent and absence of a caregiver to sign the informed consent in case of patient’s inability.

This study was conducted in accordance with the International Guidelines for Good Clinical Practice and the Declaration of Helsinki, and all subjects or their legal caregiver gave written informed consent before participation. The study obtained the approval of the Institutional Ethics Committee (see Institutional Review Board Statement). The trial has been registered in the public National Clinical Trial registry with the number NCT n. 05179330.

### 2.1. The Robotic Device

OMEGO^®^ (Tyromotion GmbH, Graz, Austria) is a robotic-assisted device, designed for the treatment of patients with lower limb impairment. It consists of a cycle ergometer with a large 2D, 24” display that provides visual feedback in a semi-immersive VR modality. Three different operating modes (passive, active motor-assisted, and fully active) are available to adapt the training to the muscle activity of the patient or according to the therapist’s choice. A computer software also allows the patient to select betwDeeen different training programs. Safe motor protection functions are also present; the foot cups are padded and non-slip, with an additional protective edge, that eliminates the risk of contact of the ankle with the pedal axis. A stable and padded grab handle performs the dual function of providing a secure grip in case of need during the exercises and a towing handle for carrying the device around. Leg guides with calf pads are also present. Two separate drives allow the mobilization of the patient in an effortless, isolated and focused manner, turning it into the missing link to gait therapy.

The cycling of the user is transmitted to a computer system, which displays the live motion of an avatar in a virtual environment. At the end of the training, the computer shows a performance analysis with a series of parameters, such as the time elapsed, the force applied by the participant on the pedals during the cycling and the combination between the two limbs.

We can consider Omego^®^ as a semi-immersive VR system according to Milgram and Kishino’s taxonomy of mixed reality [28], recently reviewed by Huygelier and colleagues [29]. This technology uses 2D displays with a limited field of view. The perspective of the environment does not change with the movements of the head but with the interaction of lower limbs. An analogous system has also been tested in a small sample of stroke patients [30].

### 2.2. Study Design

**Figure 1:**
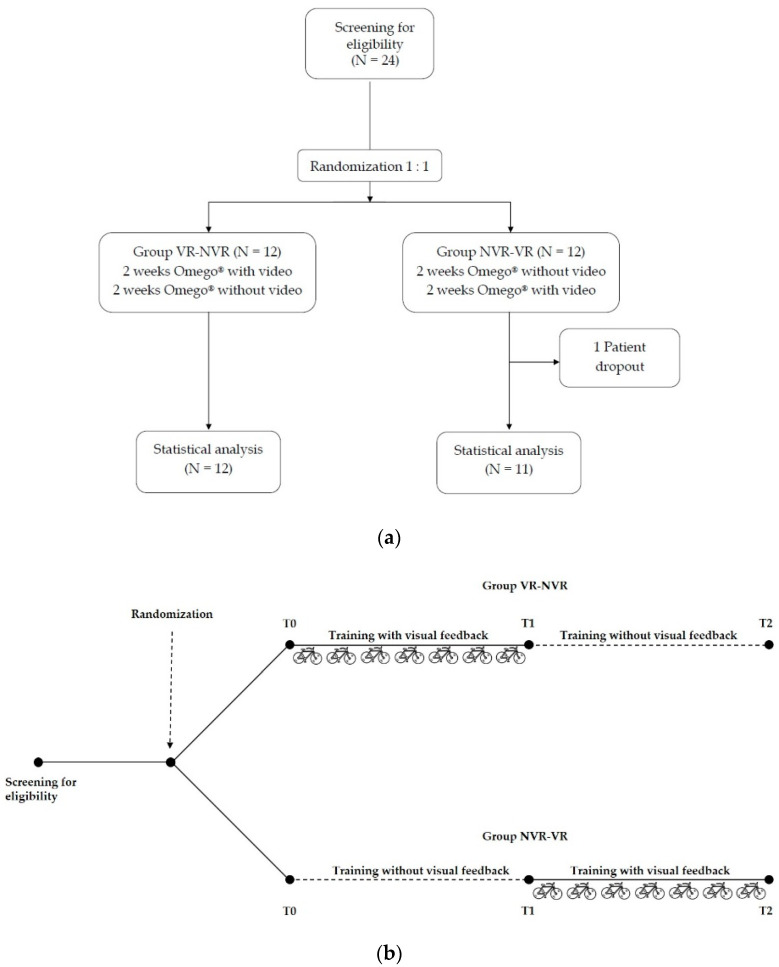
Design of the study. (**a**) Workflow of the study. (**b**) Crossover diagram of the study; VR-NVR: Virtual Reality-Non-Virtual Reality. NVR-VR: Non-Virtual Reality-Virtual Reality.

Eligible patients were randomly assigned in a 1:1 ratio to two groups by a computer-generated list. A crossover study design was adopted to make each subject as their own control. A group of patients underwent a 2-week rehabilitation training with Omego^®^ with visual feedback, followed by a 2-week training without visual feedback (Group VR-NVR). In contrast, the other group performed a 2-week rehabilitation training with Omego^®^ without visual feedback, followed by 2 weeks with visual feedback (Group NVR-VR) (Figure 1a,b).

Each patient was asked to perform a daily session of 30 min of “Biking–Landscape”. During the sessions with visual feedback, the patients performed the cycling activity by watching, in first-person point of view, the avatar of a bicycle riding through a virtual landscape. In the sessions without visual feedback, the patients performed the cycling exercise with the screen turned off.

Experimental intervention was part of the inpatient daily rehabilitation. Besides the training with Omego^®^, each patient performed 1 h of motor rehabilitation on upper limb and gait recovery. Intensity and type of movements were tailored to the patient’s residual ability. No other instrumental therapy was administered during the study.

### 2.3. Assessments

A three-step assessment was performed before starting the treatment with Omego^®^ (T0), at the end of the second week (T1), and at the end of the fourth week of training (T2). For each patient, the following outcome measures, reflecting impairments and global disability in the ICF-based model of assessment [31], were collected: (i) Level of Cognitive Functioning (LCF), as the main outcome measure for the primary aim; (ii) Disability Rating Scale (DRS) and (iii) Motricity Index for Lower Limb (MI-LL) in the most affected limb for the secondary aim.

LCF is a scale of assessment of the level of cognitive and behavioral function [32]. It includes eight rating levels, ranging from “no response” to “purposeful appropriate response”, based on the type, nature, and quality of a patient’s behavioral response. This scale has demonstrated good test–retest and inter-rater reliability as well as good concurrent and predictive validity [33].

DRS is a 30-point scale that provides information on the level of disability of patients with ABI [34]. It includes an 8-item measure, addressing all the functioning categories proposed by the World Health Organization (body function, activity, and participation). The first three items of DRS (eye opening, communication ability, and motor response) reflect body function, while the following three items (cognitive ability for feeding, toileting, and grooming) are related to cognitive function. The last two items (level of functioning and employability) reflect the participation ability of the patient. DRS has been recommended as a primary outcome measure for clinical trials involving individuals with brain injury [35,36]. Low scores reflect a better autonomy in daily life tasks.

Finally, MI is an ordinal scale that evaluates the motor and functional skills of the upper and lower limbs (in our case, only for LL) [37]. It assesses general motor function through the analysis of the essential movements of the main joints of the limb as representative of general strength. For LL, the analyzed movements are hip flexion, knee extension and ankle dorsiflexion. This scale was proven to predict the mobility outcome in post-stroke individuals [38].

### 2.4. Statistical Analysis

Being a pilot study, we selected a sample size of 12 patients per group [39]. The normality of data distribution was tested using the Kolmogorov–Smirnov test and Shapiro–Wilk test. To assess the effect of intragroup treatment, the Wilcoxon test was used, and to assess the intergroup effect, the Mann–Whitney U test was performed. Lastly, for the evaluation of the time factor, Friedman’s ANOVA was used. For post hoc analyses, Bonferroni’s correction was applied.

The Wilcoxon signed-rank test was used to compare the results obtained from the two groups after performing the same training program with and without visual feedback. Specifically, we compared the changes from baseline obtained during the robotic training with and without VR, independently from the order they were performed.

The statistical analysis was performed with IBM^®^ SPSS software (version 26.0 Armonk, NY, USA) and the statistical significance was set at *p* < 0.05.

## 3. Results

A total of 24 patients affected by severe ABI were enrolled from our high intensity neurorehabilitation ward. One patient did not complete the rehabilitation program due to a worsening of the clinical condition and was considered a dropout. Table 1 shows the demographic and baseline clinical data of the patients enrolled in the study.

Regarding time passed since the acute event, the VR-NVR group showed an average of 20.7 ± 11.5 months while NVR-VR showed an average of 39.6 ± 27.4 months, but the difference was found to be not statistically significant. Table 2 reports the ANOVA statistical analysis results for the entire sample and the post hoc analysis for the two groups (Group VR-NVR and Group NVR-VR). Considering the whole sample, a significant improvement in all outcomes was observed. In particular, significant improvements were found for the results obtained between T2 and T0 and between T1 and T0 for all outcome measures. Only for MI-LL was there a significant difference observed between T2 and T1.

When analyzing the results group by group, analogous results were found in the VR-NVR group. In particular, a statistically significant improvement was present for all the outcome measures between T2 and T0 and between T1 and T0. No significant difference was found between T2 and T1 for DRS and LCF, while a statistical difference was found for MI-LL.

Conversely, in the NVR-VR group, no significant difference was found in DRS and LCF after training without visual feedback between T1 and T0 and with visual feedback between T2 and T1. However, similarly to the other group and to the whole sample, MI-LL results showed a statistical difference.

**Figure 2:**
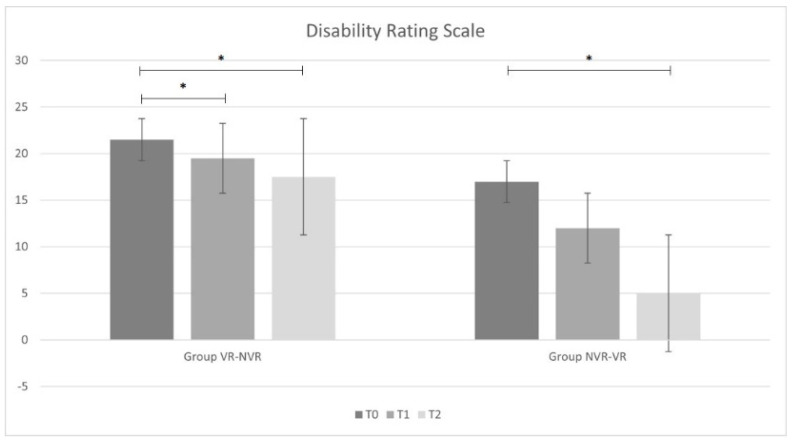
Disability Rating Scale results (mean, Standard Deviation) for Virtual Reality-Non-Virtual Reality (VR-NVR) group and Non-Virtual Reality-Virtual Reality (NVR-VR); * indicates the significant statistical differences.

**Figure 3:**
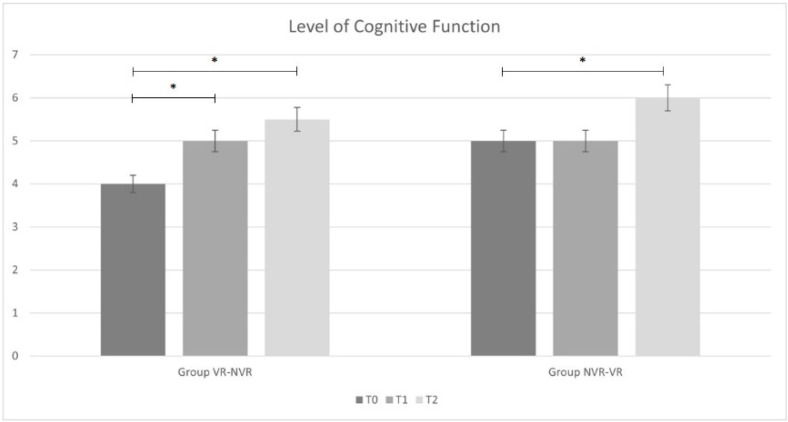
Lever of Cognitive Functioning (LCF) results (mean, Standard Deviation) for Virtual Reality-Non-Virtual Reality (VR-NVR) group and Non-Virtual Reality-Virtual Reality (NVR-VR); * indicates the significant statistical differences.

**Figure 4:**
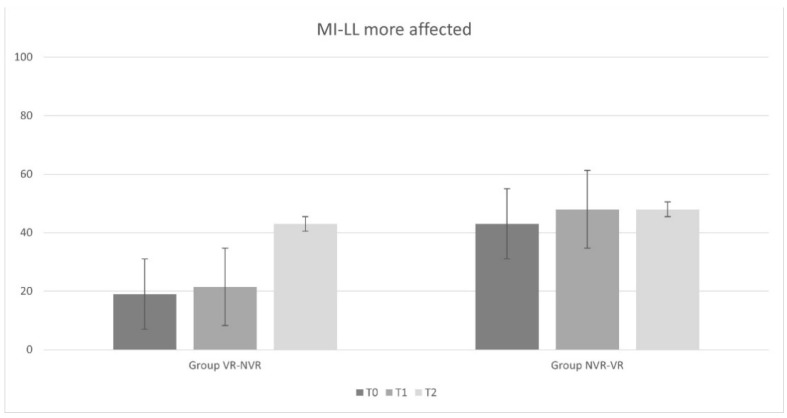
Motricity Index for Lower Limb (MI-LL) results for the most affected side for Virtual Reality-Non-Virtual Reality (VR-NVR) group and Non-Virtual Reality-Virtual Reality (NVR-VR).

All results are displayed in Figure 2, Figure 3 and Figure 4.

Considering the changes from baseline of the main outcome measures, a statistically significant difference was found in the LCF score between VR treatment and treatment without VR (*p* = 0.028). We did not find analogous results in terms of disability (DRS, *p* = 0.084) and muscle strength (MI-LL, *p* = 0.242).

## 4. Discussion

This is a pilot study aimed to evaluate whether a robotic treatment with VR feedback is more effective than standard robotic treatment in the rehabilitation of patients with severe ABI. We hypothesized that a semi-immersive video-enriched robotic system could enhance the recovery of lower limb function and reduce the overall disability of patients with severe ABI.

The results of this pilot trial support this hypothesis. After 4 weeks of training, both groups (VR-NVR and NVR-VR) significantly improved their functional impairment. The VR-NVR group showed a significant improvement in all the scales at T1. In contrast, the NVR-VR group, which was initially treated with the screen switched off, only showed a significant improvement in MI.

Our data seem to indicate that VR induced an early process of functional recovery, especially cognitive. The integration of visual feedback with muscular activation may have enhanced the stimulation of the spared cortical areas, facilitating the process of recovery. Moreover, the VR may have contributed to facilitating awareness of the performance by the patients, stimulating mental concentration and engagement, which are all elements necessary for motor learning. In this sense, type and intensity of the exercise and environmental context are important factors [40,41].

VR has been described as a computer-generated interactive virtual world that simulates the real world [29]. VR experience determines a complex psychological feeling of “being there” as well as the possibility to interact and react as if the user is in the real world. Even if we did not evaluate this aspect in the present study, we believe that our results were obtained due to a high level of active participation of the patients during the training sessions. It is also possible that our patients could have felt a reduction in psychophysiological stress during the treatment. The medium effect and a large contact area of treatment (in our case, the VR tool) could be useful in proposing sensory stimulation that results in a comfortable session of treatment [42,43].

Our results could help in the choosing the correct timing of VR rehabilitation application. As mentioned before, besides for cognitive functions, overall disability and functional impairment also significantly improved in the two groups. The use of these techniques can successfully supplement the traditional methods of treatment in the rehabilitation of patients with sABI. The use of VR in addition to conventional neuromotor rehabilitation can provide an optimal synergy for improving LL impairment [44], facilitating the functional aspects of gait [5,45], balance, and physical fitness [46]. Data regarding VR rehabilitation clinical effectiveness are currently limited due to the existence of multiple lower quality studies influencing the findings and the related conclusions [24]. It is possible that the combination of a specific motor treatment with a cognitive task could be the reason for the significant results obtained in this study. The recovery of cognitive function represents a fundamental process for all functional recoveries of these patients. At the same time, a valid cognitive function is also needed to interact with a virtual environment, which can be a limitation for certain patients with severe ABI. In this sense, the artificial environment offers the advantage of being easily modifiable to adapt to the patients’ clinical characteristics in several diseases [20,47,48,49].

Integration of visual feedback during robotic training can improve motivation and concentration compared to treatment without any feedback [50]. Due to the nature of VR training, we believe that our positive results obtained in a short period of training were due to a high level of active participation of patients during sessions, also in consideration of the possible comfort of the specific treatment [42]. We did not evaluate these outcomes, because our patients did not achieve a level of consciousness high enough to give an appropriate answer. In the next studies, it would be interesting to assess the engagement of these patients during training [51,52].

Our findings need to be assessed taking into account some limitations. Being a pilot study, our results should be considered with caution, as preliminary data and more are necessary to confirm our hypothesis. The main limit of our study is related to the small sample size, which was due to the strict inclusion/exclusion criteria. A wide range of disease severity exists between patients, particularly in terms of disability (DRS) and muscle strength (MI-LL). However, these issues are common in patients with severe ABI and cannot be easily avoided. The cross-over study design of our study reduces the impact of the small sample size and the influence of confounding factors, with each patient as his own control, making the statistical analyses more efficient. Finally, we developed an assisted training with a 30 min continuous workout. However, it may represent cardiopulmonary effort for the patients. The robotic system adjusted the force of the lower limb muscles during cycling in order to prevent a rise in cardiovascular activity. In our trial, we did not assess full active training duration and, specifically, cardiovascular function. The robotic system adapted the motor efforts during training. It is important to monitor the cardiovascular and cardiorespiratory functions of these patients due to a possible increase during VR treatments [53].

## 5. Conclusions

In conclusion, our study demonstrates that the combination of lower limb robotic training and virtual reality is an effective method to enhance lower limb functional recovery in patients with severe acquired brain injury. In particular, a significant improvement was found more quickly in patients who immediately performed the treatment of VR session with respect to those who did not practice VR. These results could add scientific data to implement research in rehabilitation with VR and robotic systems. Additional randomized trials should test the effectiveness of VR rehabilitation training on a larger population sample for various levels of disability and in domestic contexts after hospital discharge.

## Figures and Tables

**Table 1:** Demographic and clinical assessment of sample at baseline.

		Group VR-NVR (*n* = 12)	Group NVR-VR (*n* = 11)
**Gender**	F:M	4:8	5:6
**Age**	mean ± SD	54.7 ± 19.5	58.6 ± 22.6
**Time from event to rehab**	mean ± SD	20.7 ± 11.5	39.6 ± 27.4
**TBI vs. no TBI**		2:10	3:8
**DRS baseline**	Median (I, III IQR)	21.5 (19, 23)	17 (14, 18.5)
**LCF baseline**	Median (I, III IQR)	4 (4,5)	5 (4,6)
**MI More Affected baseline**	Median (I, III IQR)	19 (19, 38)	43 (33, 43)

VR-NVR: Virtual Reality-Non-Virtual Reality. NVR-VR: Non-Virtual Reality-Virtual Reality. TBI: Traumatic Brain Injury.

**Table 2:** Results of the Friedman’s ANOVA and post hoc analyses (Bonferroni’s correction) for the whole sample and individually from Group VR-NVR and Group NVR-VR individuals.

	Main			T1–T0			T2–T1			T2–T0		
	DRS	LCF	MI-LL	DRS	LCF	MI-LL	DRS	LCF	MI-LL	DRS	LCF	MI-LL
**Whole Sample**	***p* < 0.001**	***p* < 0.001**	***p* < 0.001**	***p* = 0.005**	***p* = 0.024**	***p* < 0.001**	*p* = 0.117	*p* = 0.081	***p* = 0.001**	***p* < 0.001**	***p* < 0.001**	***p* < 0.001**
**Group VR-NVR**	***p* < 0.001**	***p* < 0.001**	***p* < 0.001**	***p* = 0.043**	***p* = 0.024**	***p* < 0.001**	*p* = 0.199	*p* = 0.459	***p* = 0.005**	***p* < 0.001**	***p* < 0.001**	***p* = 0.033**
**Group NVR-VR**	***p* = 0.005**	***p* = 0.001**	***p* = 0.001**	*p* = 0.128	*p* = 0.859	***p* = 0.005**	*p* = 0.859	*p* = 0.264	***p* = 0.025**	***p* = 0.006**	***p* = 0.017**	***p* = 0.005**

VR-NVR: Virtual Reality-Non-Virtual Reality. NVR-VR: Non-Virtual Reality-Virtual Reality. DRS: Disability Rating Scale. LCF: Level of Cognitive Functioning. MI-LL: Motricity Index for Lower Limb.

## Data Availability

Data supporting the results are not available.
